# Validation of a Cognitive Self-Assessment Tool Simulating Japan's Official Digital Test for Older Drivers

**DOI:** 10.7759/cureus.93231

**Published:** 2025-09-25

**Authors:** Takayuki Asano, Asako Yasuda, Setsuo Kinoshita, Makoto Nakane, Akira Homma

**Affiliations:** 1 Research and Development, Nippontect Systems Co. Ltd., Tokyo, JPN; 2 Neurosurgery, Teikyo University Mizonokuchi Hospital, Kawasaki, JPN; 3 Psychiatry, Kinosaki Hospital, Chiba, JPN

**Keywords:** automated scoring, cognitive assessment, dementia, digital health, older drivers, screening

## Abstract

Background

A mandatory tablet-based cognitive function test for older drivers in Japan is employed for formal assessment only, terminating once a passing score is achieved and precluding a complete assessment. To bridge this gap between formal assessment and the need for self-preparation among older drivers, Nippontect Systems Co., Ltd., Japan, developed “MOGI, ” a tablet-based application that allows users to experience the entire official test for self-assessment purposes. The objective of this study was to validate “MOGI” by examining its correlation with the Mini-Mental State Examination-Japanese version (MMSE-J).

Methods

We conducted a cross-sectional study at the Minato City Silver Human Resources Center in Tokyo and among outpatients at the Oyama Orthopedics and Internal Medicine Clinic in Tochigi Prefecture. The required sample size was calculated by assuming a specific correlation coefficient, significance level, and power. Community-dwelling volunteers and individuals clinically diagnosed with mild cognitive impairment (MCI) or mild-to-moderate dementia participated from February 3 to 17, 2025. All diagnoses were made by a neurologist based on criteria from the Diagnostic and Statistical Manual of Mental Disorders, 5th Edition. Participants completed both the “MOGI” application, assessing memory and judgment via cued recall and time orientation tasks, and the MMSE-J. The agreement between automated scoring by “MOGI” and manual scoring by qualified staff was evaluated using the intraclass correlation coefficient (ICC). Spearman’s rank correlation was used to examine the relationship between “MOGI” and MMSE-J scores, and differences in “MOGI” scores among MMSE-J-based groups (≥28, 24-27, and ≤23) were evaluated.

Results

The required sample size was 37, assuming a 0.5 correlation coefficient, 5% significance level, and 90% power. A total of 42 participants, including 17 male and 25 female participants, were included in the final analysis; their mean age was 76.4±8.2 years. Excellent agreement was observed between the automated and manual scoring systems (ICC = 0.97, 95% CI: 0.94-0.98). A significant, strong positive correlation was observed between the “MOGI” total score and the MMSE-J score (ρ = 0.64, *p*<0.001). “MOGI” also demonstrated excellent discriminative ability, with significant differences in scores among the three MMSE-J-based groups (*p*<0.001 among the three groups; *p*<0.05 for ≥28 vs. 24-27; *p*<0.001 for ≥28 vs. ≤23; *p*<0.01 for 24-27 vs. ≤23).

Conclusion

“MOGI” exhibits robust validity as a cognitive assessment tool, supported by a reliable automated scoring system. By providing a comprehensive assessment experience unavailable in the official test, “MOGI” serves as a valuable complementary tool for practice, self-monitoring, and a more nuanced understanding of one's cognitive function, potentially contributing to the early detection of cognitive decline.

## Introduction

The growing prevalence of dementia has become a major global social issue and is strongly influenced by the aging population [1]. Cognitive decline not only compromises quality of life but also substantially impairs driving ability, posing a serious threat to public safety [2]. To address this concern, numerous countries have instituted or are discussing cognitive assessments of older drivers, with individual countries implementing their own systems [3-6]. In Japan, a revision of the Road Traffic Act in 2017 mandated that drivers aged 75 years or older undergo cognitive function tests when renewing their driving licenses [7]. This regulation serves as a critical public health intervention aimed at the early detection of dementia [8]. This test was designed to assess the current state of older drivers' memories and judgments to support safe driving [9]. Developed with reference to the seven-minute neurocognitive screening battery [10], it comprises tasks that assess time orientation and cued recall. Based on these results, the system aims to ensure the safety of older drivers by directing them toward appropriate senior driving courses or, when necessary, recommending specialist medical consultations. The digitization of the official test was initiated in 2022, with the tablet-based “MENKYO” system [11], developed by Nippontect Systems Co., Ltd., Japan, introduced in certain regions.

However, the use of the official digital test “MENKYO” is strictly limited to the actual examination setting, with no opportunity for older adults to practice in advance. In areas lacking adequate public transportation, automobiles are the main means of transportation. Thus, maintaining a driver’s license is essential for many older individuals. Under these circumstances, there is a growing need among older adults to familiarize themselves with the test format and understand their cognitive function beforehand. An additional, more critical limitation is that the “MENKYO” system is designed to automatically terminate the test once the user achieves a passing score. Although this system is highly efficient in determining the pass/fail status, it can conceal performance differences among older adults with more cognitive capability.

To fill this distinct gap in practice opportunities and incomplete assessment experience, Nippontect Systems Co., Ltd., Japan, has developed a new tablet-based cognitive function assessment app called “MOGI” [12]. As a key feature, “MOGI” allows the user to experience all of the same tasks and procedures available in the official “MENKYO” test, from start to finish. Even if they surpass the threshold, users are not interrupted and permitted to complete all tasks, thus providing a more comprehensive picture of their cognitive functions. Consequently, “MOGI” functions not merely as a “mock exam” but also as a tool for objective self-monitoring of one’s cognitive status.

The Mini-Mental State Examination (MMSE) is a widely used dementia screening test [13]. It assesses orientation, registration, short-term recall, attention and calculation, language, and praxis, and it is currently available in many languages [14]. Several Japanese versions of the MMSE have also been developed [15,16]. The Mini-Mental State Examination-Japanese version (MMSE-J) has been created to faithfully translate the original version and appropriately adapt it to Japanese culture [16]. It is widely used in clinical and research settings as a dementia screening tool.

This study aimed to validate “MOGI” by examining its correlation with the MMSE-J and evaluating whether MMSE-J stratification is reflected in “MOGI” scores.

## Materials and methods

Study design and participants

Participants were recruited from registered members of the Minato City Silver Human Resources Center in Tokyo and outpatients of the Oyama Orthopedics and Internal Medicine Clinic in Tochigi Prefecture. Data were collected from February 3 to 17, 2025.

At Oyama Clinic, participants included individuals diagnosed with mild cognitive impairment (MCI) or mild-to-moderate dementia. All diagnoses were made by a neurologist based on the criteria from the Diagnostic and Statistical Manual of Mental Disorders, 5th Edition [17].

The required sample size was calculated to be 37, assuming a correlation coefficient of 0.5, a significance level of 5%, and a power of 90%. In total, 42 individuals participated in the study, which exceeded the calculated requirement.

Ethical considerations

This study was conducted in accordance with the ethical principles of the Declaration of Helsinki, and the study protocol was approved by the Asai Dermatology Institutional Review Board (January 20, 2025; No. 2025012001). All participants were provided with sufficient explanation of the study objectives, procedures, anticipated burdens, and benefits. Subsequently, signed informed consent was obtained from each participant. Participants’ data were anonymized at the time of collection, and privacy was scrupulously protected.

Assessments

1. “MOGI” Tablet-Based Cognitive Assessment Application

“MOGI” is an iPad-based application that reliably replicates the Japanese driver’s license renewal cognitive test and comprises the following components.

Cued recall task: Participants are shown 16 illustrations on four cards and explicitly instructed to memorize them for a later recall test. After an intervening task, participants are requested to recall the items in two phases: without hints (free recall) and with hints (cued recall).

Time orientation task: Participants are requested to write about the current year, month, date, day of the week, and time.

Scoring: The participants follow audio guidance via headphones and write their answers directly on a tablet screen using a stylus pen. The handwritten input is converted into text using an online handwritten Japanese character recognition engine (iLabo Co., Ltd., Japan) [18,19] and automatically scored and tabulated based on the official guidelines of the National Police Agency [20]. The total “MOGI” score is the weighted sum of its two components, calculated as an integer with a maximum score of 100 points. The score is derived using the following formula: Total Score = round(Cued Recall Score × 2.499 + Temporal Orientation Score × 1.336). The maximum score for the cued recall task is 32, while that for the temporal orientation task is 15. In “MOGI,” individuals who score less than 36 on the total score are considered to be at risk of dementia, according to the official guidelines of the National Police Agency [20].

2. MMSE-J

The MMSE-J scores range from 0 to 30, with higher scores indicating better cognitive function. A cutoff point of 23/24 is commonly applied. The MMSE-J was used in this study under proper license.

Study procedure

Demographic information, including age, sex, and years of education, was collected from each participant. Subsequently, participants completed tests in the order of MMSE-J and “MOGI,” and the order was not randomized. For “MOGI,” participants wore headphones and operated the tablet themselves, following on-screen and audio instructions. The handwritten response data from “MOGI” was scored both by the automated system and manually by qualified staff (human scoring) to confirm the agreement between the two methods.

Statistical analysis

The intraclass correlation coefficient (ICC) was calculated to evaluate the agreement between the automated and human scoring of “MOGI” by two-way mixed-effects, absolute agreement. Non-parametric tests were selected because of the non-normal distribution of cognitive scores. For the primary analysis, Spearman's rank correlation coefficient was used to assess the relationship between the “MOGI” total score and the MMSE-J total score. Additionally, the participants were classified into three groups based on their MMSE-J scores: normal-range (≥28), suspected MCI (24-27), and suspected dementia (≤23). The Kruskal-Wallis test was employed to examine significant differences in “MOGI” scores (total, time orientation, and cued recall) among these three groups. Post-hoc pairwise comparisons were conducted using Mann-Whitney U tests with Bonferroni corrections. The level of statistical significance was set at a two-sided *p*<0.05. All analyses were performed in Python (v.3.12.2) [21].

## Results

A total of 42 participants were included in the final analysis. Their mean age was 76.4±8.2 years, with 17 male and 25 female participants. The mean duration of education was 13.8±2.8 years, and the mean MMSE-J score was 26.5±5.0 (Table 1). The ICC for agreement between the automatically scored total “MOGI” score and the manually scored total was 0.97 (95% CI: 0.94-0.98), indicating a high level of concordance. This suggested that the automated scoring system could accurately convert handwritten inputs into text with minimal errors. Subsequent analyses used the scores from the automated system.

**Table 1:** Participant demographics SD: Standard deviation; MMSE-J: Mini-Mental State Examination-Japanese version

	Total	MMSE-J-based groups		
		≥28	24-27	≤23
Participants (n)	42	28	7	7
Age, years (mean ± SD)	76.4 ± 8.2	72.9 ± 7.4	81.7 ± 4.9	85.1 ± 2.1
Education, years (mean ± SD)	13.8 ± 2.8	14.8 ± 2.3	12.6 ± 3.4	11.0 ± 1.7
MMSE-J (mean ± SD)	26.5 ± 5.0	29.2 ± 0.8	25.6 ± 1.3	16.6 ± 4.0

**Figure 1:**
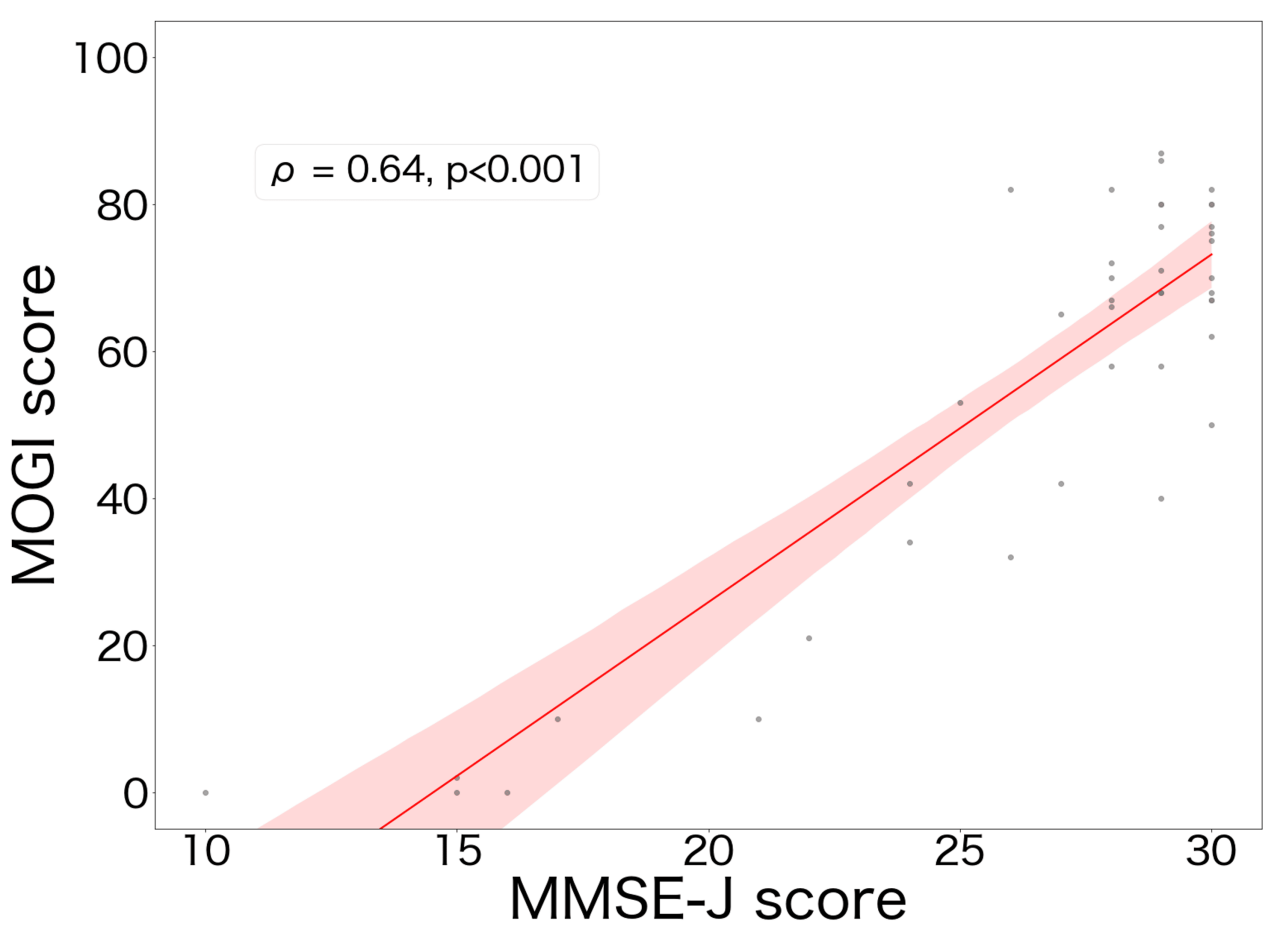
Correlation between the “MOGI” total score and the MMSE-J score This scatter plot illustrates the relationship between the total score on the “MOGI” application and the score on the MMSE-J. The solid line represents the linear regression line, and the shaded red area indicates the 95% confidence interval. MMSE-J: Mini-Mental State Examination-Japanese version

A significant, strong positive correlation was detected between the “MOGI” total score and the MMSE-J score (Spearman's ρ = 0.64, *p*<0.001) (Figure 1).

When participants were divided into three groups based on MMSE-J scores (≥28 group: n = 28; 24-27 group: n = 7; ≤23 group: n = 7), the “MOGI” total score, the time orientation score, and the cued recall score showed significant between-group differences (Kruskal-Wallis test, p<0.001 for all). Subsequent multiple comparisons revealed that both the total “MOGI” score and the cued recall score significantly differed between each pair of the three groups. However, for the time orientation task, no significant differences were observed between the normal range and suspected MCI groups, although both groups scored significantly higher than the suspected dementia group (Table 2).

**Table 2:** Comparison of “MOGI” scores across groups classified by MMSE-J score SD: Standard deviation; MMSE-J, Mini-Mental State Examination-Japanese version

		MMSE-J-based groups			P-value of Kruskal-Wallis test	P-value of Mann-Whitney U test with Bonferroni correction		
		≥28	24-27	≤23		≥28 vs. 24-27	24-27 vs. ≤23	≥28 vs. ≤23
MOGI	Total (mean ± SD)	70.9 ± 10.7	50.0 ± 18.1	6.1 ± 8.0	< 0.001	< 0.05	< 0.01	< 0.001
	Time orientation task (mean ± SD)	13.5 ± 2.4	11.1 ± 2.7	2.4 ± 2.7	< 0.001	0.091	< 0.01	< 0.001
	Cued recall task (mean ± SD)	21.3 ± 3.4	14.1 ± 6.0	1.3 ± 1.9	< 0.001	< 0.05	< 0.01	< 0.001

Furthermore, all seven participants with an MMSE-J score of ≤23 scored below 36 on “MOGI” (a score indicating risk of dementia). Conversely, the MMSE-J score classified two participants who scored below 36 on “MOGI” into the 24-27 group. These results indicate that, although the two tests are generally concordant, their evaluations may differ in certain cases. “MOGI” may be more sensitive to subtle impairments in some individuals, whereas MMSE-J may classify them as borderline or within normal limits.

## Discussion

In this study, our findings demonstrated that “MOGI,” a self-assessment tool that fully replicates Japan's official digital cognitive test, strongly correlates with the MMSE-J and that its score distribution corresponds to the cognitive levels classified by the MMSE-J. These findings suggest that “MOGI” could be a valuable cognitive screening tool.

The official “MENKYO” test, which is terminated upon reaching a passing score, fails to capture performance differences among individuals at the higher end of the cognitive spectrum, for example, distinguishing between an individual who barely passes and an individual who could achieve a perfect score. In contrast, “MOGI” requires the completion of all tasks, allowing for a higher-resolution assessment of cognitive function.

The ceiling effect in cognitive screening tests can be problematic, particularly for assessing cognitively normal individuals and those in the early stages of MCI. The MMSE, a widely used dementia screening tool, is noted for its inability to adequately capture subtle cognitive differences between healthy individuals and those with MCI, as many healthy participants achieve maximum or near-maximum scores [22,23]. The Montreal Cognitive Assessment (MoCA), developed for MCI screening [22], provides a wider distribution of total scores and attenuated ceiling effects relative to the MMSE, improving its ability to distinguish between cognitively normal individuals and those with MCI [22,24]. Nevertheless, scores among healthy populations still tend to cluster at the upper end of the scale [25]. Computerized cognitive assessment batteries, such as Cambridge Neuropsychological Test Automated Battery (CANTAB) [26] and CogState [27], are designed to minimize ceiling effects by incorporating metrics like reaction time and are utilized for screening not only dementia but also MCI [28,29]. Consistent with these established digital tools, “MOGI” demonstrated no ceiling effect in this study. A particularly noteworthy finding was the wide dispersion of “MOGI” scores even among participants who achieved a maximum score of 30 on the MMSE-J. Although “MOGI” was developed to screen for dementia-level cognitive decline, this result suggests that “MOGI” has the potential to detect cognitive decline equivalent to MCI that is undetectable by the MMSE.

A key feature of “MOGI” is that it allows individuals to take the test independently on a tablet by following automated audio guidance. If older adults can use “MOGI” for practice and self-assessment at home or in community settings, it could alleviate anxiety regarding the official license renewal test, as well as contribute to regular self-monitoring.

Nevertheless, the evaluations for suspected dementia did not perfectly align between the “MOGI” and the MMSE-J. Although “MOGI” formally evaluates two primary domains (temporal orientation and memory), its testing process incorporates more complex tasks, such as visual recognition and handwritten responses. Consequently, while both tests assess overlapping cognitive functions, “MOGI” may tap into different processes, such as the purer extent of memory storage and the integrity of visuo-motor integration. Therefore, the inconsistencies in classification could stem not only from these fundamental differences in test items and formats but also from technical factors, such as potential misinterpretations of handwritten inputs by the character recognition engine.

This study had several limitations. First, the relatively small sample size and recruitment from limited sources may restrict the generalizability of the findings. Second, the cross-sectional design did not allow the prediction of future “MOGI” scores. Therefore, future research is required to build on these initial findings. Longitudinal studies are necessary to determine whether changes in “MOGI” scores can predict an increased risk of cognitive impairment and driving safety concerns, as well as to investigate potential learning and practice effects from repeated administration. Future studies should focus on larger and more diverse populations and validate other cognitive measures, such as the MoCA and clinical diagnosis. Finally, dedicated studies are required to evaluate the usability of the tool, consider the influence of digital literacy on performance, and conduct a more rigorous validation of the scoring threshold to establish optimal cutoff points.

Nevertheless, unlike “MENKYO,” which can only be used for the official test at license renewal, “MOGI” has broad applicability as both a practice tool and a self-assessment resource. In the future, its widespread introduction into care facilities for older individuals, community health programs, and in-home care could empower older adults to regularly monitor changes in their cognitive function, paving the way for early intervention and safety measures in their daily lives.

## Conclusions

This study demonstrated that the tablet-based cognitive test application “MOGI” is a valid cognitive assessment tool that strongly correlates with the MMSE-J. By offering a “practice opportunity” and a “complete assessment experience” - features absent in the official test - “MOGI” enables older adults to understand their cognitive function more deeply and proactively. The tool holds considerable promise as a practical aid for driver's license renewal, as well as a potentially valuable and accessible means for self-monitoring and early detection of cognitive decline in an aging society.
